# Adult thoracic empyema: A comparative analysis of tuberculous and nontuberculous etiology in 75 patients

**DOI:** 10.4103/0970-2113.71939

**Published:** 2010

**Authors:** Somenath Kundu, Subhra Mitra, Subhasis Mukherjee, Soumya Das

**Affiliations:** *Department of Respiratory Medicine, IPGME&R and SSKM Hospital, Kolkata - 700 020, India*; 1 *Department of Respiratory Medicine, Midnapore Medical College and Hospital, West Midnapore, India*; 2 *Department of Respiratory Medicine, R.G. Kar Medical College & Hospital, Kolkata - 700 004, India*; 3 *Department of Respiratory Medicine, BS Medical College & Hospital, Bankura - 700 020, West Bengal, India*

**Keywords:** Bronchopleural fistula, empyema, parapneumonic effusion, tuberculous empyema

## Abstract

**Background:**

Thoracic empyema is a disease of significant morbidity and mortality, especially in the developing world where tuberculosis remains a common cause. Clinical outcomes in tuberculous empyema are complicated by the presence of concomitant fibrocavitary parenchymal disease and frequent bronchopleural fistulae. We performed a prospective study over a one-and-a-half-year period with the objective of comparing the clinical profiles and outcomes of patients with tuberculous and nontuberculous empyema.

**Materials and Methods:**

A prospective study of adult cases of nonsurgical thoracic empyema admitted in a tertiary care hospital in eastern India was performed over a period of 18 months. A comparative analysis of clinical characteristics, treatment modalities, and outcomes of patients with tuberculous and nontuberculous empyema was carried out.

**Results:**

Seventy-five cases of empyema were seen during the study period, of which 46 (61.3%) were of nontuberculous etiology while tuberculosis constituted 29 (38.7%) cases. Among the nontuberculous empyema patients, *Staphylococcus aureus* (11, 23.93%) was the most frequent pathogen isolated, followed by Gram-negative bacilli. Tuberculous empyema was more frequent in younger population compared to nontuberculous empyema (mean age of 32.7 years vs. 46.5 years). Duration of illness and mean duration of chest tube drainage were longer (48.7 vs. 23.2 days) in patients with tuberculous empyema. Also the presence of parenchymal lesions and bronchopleural fistula often requiring surgical drainage procedures was more in tuberculous empyema patients.

**Conclusion:**

Tuberculous empyema remains a common cause of empyema thoracis in a country like India. Tuberculous empyema differs from nontuberculous empyema in the age profile, clinical presentation, management issues, and has a significantly poorer outcome.

## INTRODUCTION

In developed countries nonmycobacterial pulmonary infections and surgical procedures constitute the majority of thoracic empyema cases,[1] whereas in the developing world tuberculosis accounts for a sizeable number.[2] Clinical outcomes of tuberculous empyema are generally believed to be worse compared to those of nontuberculous empyema because of protracted illness, presence of concomitant fibrocavitary lung lesions, high bacillary load, development of bronchopleural fistulae (BPF), and requirement for complicated thoracic surgeries in the face of compromised lung function. Against this background, the present study was conducted to compare the clinical characteristics and outcome of patients with tuberculous and nontuberculous empyema.

## MATERIALS AND METHODS

### Study design

This study was a prospective analysis of all adult cases of nonsurgical thoracic empyema admitted in the department of respiratory medicine of a tertiary care teaching hospital in eastern India over a period of 18 months (Dec 2005–May 2007).

### Patient selection

#### Case definition

Thoracic empyema was defined as pleural effusion that fulfilled at least one of the following criteria: (1) the presence of frank pus on pleural aspiration; (2) presence of organism on pleural fluid culture; (3) positive pleural fluid Gram stain. Tuberculous empyema was defined as cases of thoracic empyema with one of the following: (1) pleural fluid smear positive for acid fast bacilli (AFB); (2) sputum positive for AFB and having radiological lesions consistent with active parenchymal tuberculosis on chest x-ray/CT scan of the thorax (nodular consolidation with or without cavity in apex, tree in bud appearance). Probable tuberculous empyema was defined as empyema in patients who had radiological evidence of active pulmonary tuberculosis on chest x-ray/CT scan of the thorax or were sputum positive for AFB.[2] Written informed consent was taken from all patients and the study was cleared by the institute’s ethics committee.

#### Exclusion criteria

(1) Age less than 15 years; (2) empyema secondary to penetrating or blunt chest trauma; (3) empyema secondary to any surgical procedure.

### Study protocol

Detailed demographic and clinical parameters including age, sex, symptom duration (fever, weight loss, cough, sputum, hemoptysis, shortness of breath, chest pain) were evaluated in all patients fulfilling the case definition. Presence of any comorbidities like diabetes mellitus, HIV infection, seizure disorder, liver abscess, rheumatoid arthritis, and malignancy was documented. Chest radiographs were obtained in all patients at the admission, after intercostal tube drain (ICTD) insertion, ICTD removal and at discharge, while ultrasound (USG) and computed tomography (CT) of chest were done if necessary. Pleural fluid was collected aseptically by thoracentesis and if macroscopically purulent was submitted for Gram stain, culture (aerobic), and smear for AFB. Nonpurulent fluid was studied additionally for total leukocyte count (TLC), differential leukocyte count (DLC), protein, sugar, and LDH. Anaerobic culture was carried out if there seemed a suspicion of anaerobic empyema (in those with h/o aspiration, alcoholism, seizure, periodontal disease). Mycobacterial culture of pleural fluid was not done in this study owing to nonavailability of BACTEC system and facilities for drug-sensitivity testing. We were also influenced by previous studies[2] which showed technical problems causing low yield of culture of *Mycobacterium tuberculosis* from empyema fluids. Complete blood counts, renal and liver function tests, blood for HIV serology, blood sugar (fasting and postprandial), and sputum for AFB smear were sent in for all patients.

Etiology of empyema was decided based on history, physical examination, radiology, and empyema fluid analysis. BPF was diagnosed if chest X-ray prior to thoracentesis revealed horizontal air fluid level in the upright position and air leak through the tube thoracostomy persisted for more than 24 hours after tube thoracostomy.

Closed thoracostomy (intercostal tube drainage, ICTD) was carried out with a straight chest tube (Romson 28–32 F) attached to a water-seal drainage system or by the placement of pigtail catheters (8.5 Fr) under ultrasound guidance when fluid was loculated. Continuous drainage was maintained until fluid was serous, daily collection was less than 50 ml, pleural cavity was obliterated by expansion of the lung, and any BPF was sealed. In cases of multiloculated empyemas where pigtail catheter insertion was not possible, serial ultrasound-guided aspiration of pleural fluid was done. All patients with nontuberculous empyema received antibiotics for a total duration of 4–6 weeks depending on clinical response. For the initial 14 days, intravenous antibiotics were administered followed by oral antibiotics. Empirical antibiotics chosen in this study were a third-generation cephalosporin plus clindamycin or metronidazole for anaerobic coverage. Antibiotic regimen was changed subsequently if culture sensitivity reported resistant organisms. Aminoglycosides were not used because of their low concentration in pus. All patients of tuberculous empyema received category I or category II antituberculous drugs (ATDs) treatment under DOTS strategy of WHO.[3] In addition, they were initially put on intravenous antibiotics further course of which was determined by Gram stain/culture report and clinical response. Use of intrapleural fibrinolytics was not included in the study as MIST trial has hinted at its equivocal role.[4] In those patients who failed to respond to antibiotic therapy and ICTD (after checking for clogged tubes, incorrect tube placement) as evidenced by the persistence of fever or leukocytosis due to loculations or inadequate drainage, nonexpansion of the lung or the presence of BPF, surgical drainage was carried out in the cardiothoracic department of the institute. Decortication, decortication with closure of BPF using intercostal muscle flap, and thoracoplasty were the three surgical procedures performed.

### Outcome

Allpatients were followed up for a minimum period of 6 months. Outcome was defined as one of the following:

#### Cure

Complete resolution of symptoms, normalization of laboratory markers of infection/inflammation, and complete lung expansion with residual pleural thickening of <2 cm in chest X-ray PA.

#### Failure

Recurrence or persistence of BPF after medical and surgical management.

#### Death

During the course of illness due to the disease process.

### Statistical analysis

Statistical analyses were performed using SPSS version 10.0 (SPSS inc., Chicago, IL) software for MS-Windows. Descriptive frequencies were expressed using mean. *P* value was calculated using Fisher’s exact test of significance for categorical variables and independent sample *t* test for continuous variables, and *P* value <0.05 was considered to be significant. Mean, standard deviation, and ranges were also calculated where relevant.

## RESULTS

Seventy-five patients of empyema were admitted during the study period of which 29 (38.7%) were of tuberculous etiology and 46 (61.3%) were of nontuberculous etiology. Frank pus was aspirated in 21 cases of tuberculous empyema (72.4%) and 37 cases of nontuberculous empyema (80.4%). No significant difference in sex distribution was noted with male preponderance seen in both the groups (21 cases, 72.4% in tuberculous empyema and 34 cases, 73.9% in nontuberculous empyema). The majority (13 cases, 44.8%) of tuberculous empyema cases belonged to a relatively younger age group (15–30 years), whereas 52% (24 cases) of nontuberculous empyema patients were above 45 years of age. Mean age in the tuberculous empyema group was 32.7 years (range: 14–65 years) compared to mean age of 46.5 years (range: 13–72 years) in nontuberculous group. Twenty-four cases (82.8%) of tuberculous empyema had illness of more than 1 month’s duration [Table 1], whereas only 13 cases (28.3%) of nontuberculous empyema had duration of more than 1 month and mean duration of illness was significantly higher in tuberculous group (mean 171.2 days) compared to nontuberculous group (mean 20 days). There was not much difference in routine laboratory markers between the two groups, mean TLC was 10027.6/cmm, and mean ESR was 72 mm at the end of first hour in the tuberculous group, corresponding values in the nontuberculous group were 14877.1/cmm and 76 mm, respectively. All nontuberculous empyema patients had fever, which was also present in 75% of tuberculous empyema patients. Hemoptysis was more common in tuberculous compared to nontuberculous empyema (seven cases, 24.3% vs. three cases, 6.5%). There were 5 cases (17.2%) of empyema necessitans and 14 cases (48.8%) of BPF in the tuberculous empyema group compared to none and five cases (10.9%), respectively, in the nontuberculous empyema group. Diabetes mellitus, the commonest comorbid condition, was seen in 23 (30.7%) of the total cases [Table 2]. It was equally prevalent in tuberculous empyema (10 cases, 34.5%) and nontuberculous empyema (23 cases, 28.3%) patients. Other comorbidities were alcoholism in nine cases (12%), seizure disorder, HIV positivity, liver abscess, malignancy on chemotherapy, and rheumatoid arthritis [Table 2].

**Table 1:** Comparison of clinical characteristics – tuberculous vs. nontuberculous empyema

	Tuberculous empyema (*n* = 29)	Nontuberculous empyema (*n* = 46)	*P* value
Age in years mean [SD] (range)	32.7 [8.45], (14–65)	46.5 [15.38] (13–72)	
Duration of Illness more than 1 month* [mean, range]	24 (82.8) [171.2 days, 1–24 months]	13 (28.3) [20 days, 5 days to 3 months]	0.0001
Cough	24 (82.8)	38 (82.6)	0.76
Fever*	22 (75.5)	46 (100)	0.04
Shortness of breath	27 (93.1)	46 (100)	0.05
Hemoptysis*	7 (24.3)	3 (6.5)	0.05
Empyema necessitans	5 (17.2)	0	
Bronchopleural fistula (BPF)*	14 (48.8)	5 (10.9)	0.0004

* *P*<0.05; Figures in parenthesis are in percentage

**Table 2:** Comorbid conditions

Comorbidities	Tuberculous empyema (*n* = 29)	Nontuberculous empyema (*n* = 46)	Total (*n* = 75)
Diabetes mellitus	10 (34.5)	13 (28.3)	23 (30.7)
Alcoholism	1	8	9 (12)
HIV positive	1	1	2 (2.7)
Seizure disorder	0	2	2 (2.7)
Cancer/chemotherapy	0	3	3 (4)
Liver abscess	0	2	2 (2.7)
Prolonged steroid use	0	1	1 (1.3)

Figures in parenthesis are in percentage

### Radiology

**Figure 1:**
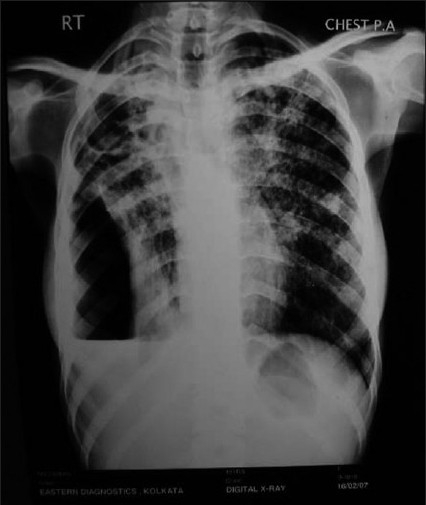
Chest X-ray showing right-sided pyopneumothorax with bilateral parenchymal lesions

Twenty patients (68.9%) with tuberculous empyema had pyopneumothorax compared to nine cases (19.6%) of nontuberculous empyema. Associated parenchymal lesion in the same or opposite lung was also significantly higher in the tubercular group (20 cases, 68.8%) [Figure 1] compared to the nontubercular group (five cases, 10.9%). Focal areas of parenchymal consolidation (13 cases, 44.8%) was the commonest lung lesion in tuberculous empyema, cavitary lesion was found in four cases (13.8%), and bilateral lesion was noted in six cases (20.7%). Associated consolidation in the ipsilateral lung parenchyma was found in only five cases (10.9%) of nontuberculous empyema.

### Bacteriologic spectrum

Among the 29 tuberculous empyema patients [Table 3], pleural fluid AFB smear was positive in 27 cases (93.1%), sputum smear for AFB was positive in 19 cases (65.5%), and both sputum and pleural fluid smear were positive in 18 cases (62.1%). Of the two patients of tuberculous empyema whose pleural fluid was negative for AFB, one was sputum smear positive for AFB with cavitary lesion on chest X-ray, while the other patient diagnosed as probable tuberculous empyema, had CT evidence of active pulmonary tuberculosis in the form of focal consolidation in the right upper lobe with areas of cavitation. Eight patients with tuberculosis also had the evidence of coexisting bacterial infection with pleural fluid for Gram-stains being positive. Among the 46 cases of the nontuberculous empyema group, pleural fluid culture was positive in 20 cases (43.5%), only Gram-stain was positive but culture was negative in additional nine cases (19.6%) and in the remaining17 patients (36.9%) the pus was sterile. Pleural fluid culture was positive in 9 of the 14 patients (64.2%) who had received antibiotics for less than 48 hours prior to the admission and in 11 of the 32 patients (34.4%) who had received a more prolonged antibiotic course prior to admission. *Staphylococcus aureus* was the commonest organism isolated (on 11 occasions), followed by Gram-negative bacilli (on 10 occasions) [Table 4]. In two patients, pleural fluid culture showed mixed growth of *Staph. aureus* and Gram-negative bacilli.

**Table 3:** Diagnosis of tuberculous empyema (*n* = 29)

AFB-positive pleural fluid smear	27 (93.1)
AFB-positive sputum smear	19 (65.5)
Parenchymal consolidation/cavity in CXR/CT	20 (68.9)

Figures in parenthesis are in percentage

**Table 4:** Bacteria isolated in pleural fluid culture of nontuberculous empyema patients (*n* = 22)

Organism	Number (%)
Gram-positive bacteria	
Staphylococcus aureus	11 (50)
Streptococcus haemoliticus	1
Gram-negative bacteria	10 (45.5)
*Pseudomonas aeruginosa*	3
*Klebsiella pneumoniae*	1
*Proteus* spp.	2
*Escherichia coli*	1
*Enterobacter* spp.	1
*Citrobacter* spp.	1
*Acinetobacter* spp.	1

### Treatment and outcome

**Figure 2:**
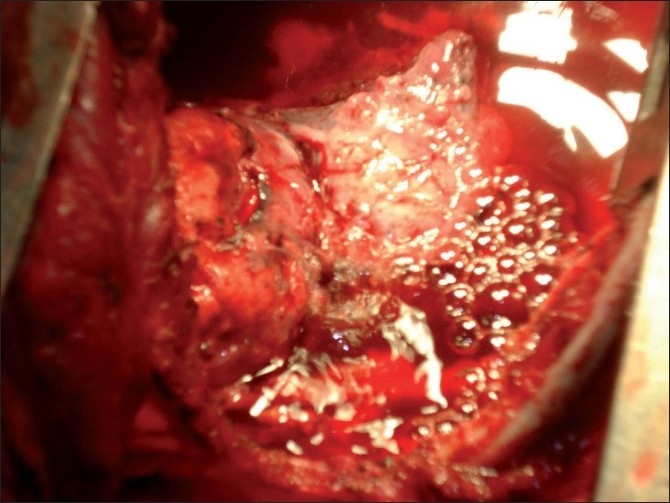
Peroperative photograph during decortication showing air bubbles from bronchopleural fistula

**Figure 3:**
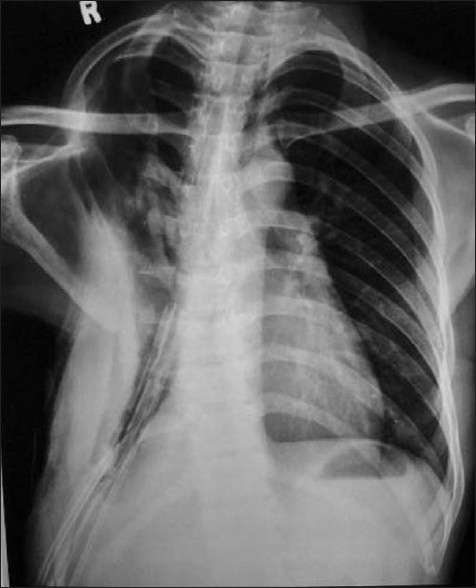
Chest X ray after thoracoplasty

The drainage modalities in nontuberculous empyema [Table 5] were intercostal tube drainage in 33 cases (71.7%), pigtail catheter drainage in 4 cases (8.7%), and serial USG-guided aspiration in 9 cases (19.6%). Ten patients with tuberculous empyema (34.5%) had a past history of intake of antitubercular drugs (ATDs), and four of them were defaulters with a history of taking ATDs from unsupervised regimens. These 10 cases were put on category II DOTS. Seventeen patients (70.8%) in the tuberculous empyema group required ICTD for more than 1 month compared to only 9 (24.3%) in the nontuberculous group; mean duration of intercostal tube drainage was also higher in the tubercular group (48.7 days) as against the nontubercular group (23.2 days). Sixteen patients (55.2%) with tuberculous empyema required surgery despite ATDs and ICTD, whereas only five (10.9%) in the nontuberculous group needed surgery. Of the 16 patients in the tuberculous empyema group who needed surgery, 4 required decortication only, 10 patients required decortication with closure of bronchochopleural fistula [Figure 2], and 2 of them needed additional thoracoplasty [Figure 3].

**Table 5:** Drainage modalities

	Tuberculous empyema (*n* = 29)	Nontuberculous empyema (*n* = 46)
USG-guided aspiration	5 (17.2)	9 (19.6)
Pigtail catheter drainage	None	4 (8.7)
ICTD	24 (82.88)	33 (71.7)
Surgical drainage*	16 (55.2)	5 (10.9)

* *P* value<0.0001; Figures in parenthesis are in percentage

The outcome of treatment was also poor in the tuberculous group [Table 6]. Overall cure of nontuberculous empyema occurred in 100% of the cases but as far as tuberculous empyema is concerned, there was failure of lung expansion even after surgery in four cases (13.8%) and in one case death occurred from extensive pulmonary parenchymal disease, and persistent pleural sepsis following bronchial stump dehiscence.

**Table 6:** Treatment modalities and outcome – tuberculous vs. nontuberculous empyema

	Tuberculous empyema (*n* = 29)	Nontuberculous empyema (*n* = 46)	*P* value
Mean duration of ICTD [range]	48.7 days [10–135 days]	23.2 days [6–67 days]	
ICTD > 1 month*	17 (70.8)	9 (24.3)	0.04
Surgical drainage*	16 (55.2)	5 (10.9)	0.0001
Failure of lung re-expansion after surgery	4 (13.8)	None	
Death	1 (3.4)	None	

* *P* value<0.05; Figures in parenthesis are in percentage

## DISCUSSION

Thoracic empyema continues to be an important cause of morbidity specially in developing countries[2] and this has been reflected in this study by the number of cases enrolled (75 over an 18 month period).Pulmonary infections (including community acquired pneumonia, bronchiectasis and lung abscess) are the commonest cause of thoracic empyema in the western countries followed by surgical trauma.[1,4] In contrast, studies from India reveal that tuberculosis accounts for a large number of empyema cases (38.6–65%).[2,5–9] In our study also, tuberculous empyema accounted for 29 of the 75 cases, i.e., 38.7% of total empyema cases.

The majority of the tuberculous empyema patients (*n* = 13, 44.8%) in our study presented at a young age (15–30 years) and mean age was 32.7 years. This finding has been supported by other Indian studies, like Goyal *et al*.[6] (median age for tuberculous empyema 21–40 years) and Acharya *et al*.[7] (median age for tuberculous empyema 20–40 years). A high incidence of pulmonary tuberculosis in this age group has been documented in the country in some studies.[10] Among nontuberculous empyema patients, pleural fluid culture was positive in 43.5% (20 cases, in two pleural fluid culture showed mixed growth), *Staph*. aureus was the commonest organism isolated (*n* = 11, 50%) followed by Gram-negative organisms collectively (*n* = 10, 45.4%). Other Indian studies have reported pleural fluid culture positivity of around 42%[5,9] with *Staphylococcus* being the commonest one.[9] Although *Streptococcus pneumoniae* is a common cause of community-acquired pneumonia, pleural fluid cultures are usually negative, whereas staphylococcal parapneumonic effusions are more likely to be culture positive.[1] This, along with prolonged prior antibiotic use (32 cases, 69.6%), was probably an important factor for pleural fluid culture negativity and absence of yield of *Strep. pneumoniae* in our cases. Also anaerobic culture performed in 20 cases of suspected anaerobic infection was negative which may be due to the stringent conditions required for isolation of anaerobes. A low yield of anaerobic organisms has been the finding of Malhotra *et al*.[2] (4 of 72 bacteria isolated). Although in the early 1970s anaerobic organisms were most frequently isolated, in the 1990s aerobic organisms are most often responsible for empyemas.[1] *Pseudomonas* spp., *Proteus* spp., *Klebsiella* spp., *Acinetobacter* spp., *Citrobacter* spp. were the Gram-negative organisms encountered in this study. Cases of Gram-positive empyema were seen in the younger age group (15–30 years) compared to 60% cases of Gram-negative empyemas in the age group of more than 60 years. Cases of Gram-negative empyemas had higher incidence of BPF (30%), prolonged intercostal tube drainage (mean duration 39.5 days), and increased requirement of surgery (30% vs. 8%) compared to Gram-positive cases, though perhaps the numbers of organisms in the two groups are too small to reach a meaningful statistical conclusion. A similar adverse outcome of Gram-negative empyema has been reported by Begany *et al*.[11] Serious associated diseases were present in 30 cases (65%) of nontuberculous empyema, lower than figures reported (82%) in adult empyema in the developed world.[12]

Regarding the diagnosis of tuberculous empyema, unlike most of the Indian studies which have revealed a low percentage of bacteriological positivity in sputum and pleural fluid,[5,9] our study has revealed a high bacteriological yield. Pleural fluid smear for AFB was positive in 93.1% (27cases) of tuberculous empyema, sputum smear was positive in 65.5% (19 cases), and both sputum and pleural fluid smear for AFB were positive in 62.1% (18 cases). Goyal *et al*.[6] also reported high pleural fluid smear positivity for AFB (71.69%), while Malhotra *et al*.[2] in their study reported smear positivity in 20 of their 41 cases of tuberculous empyema. This high percentage of pleural fluid smear positivity can be attributed to the adoption of standardized methodology for mycobacterial staining and reporting as per the DOTS strategy. From the point of view of pathogenesis, such high mycobacterial smear positivity in the sputum and pleural fluid is due to high bacillary load, which is reflected by the presence of concomitant parenchymal lesions and a high incidence of bronchopleural fistula (*n* = 14, 48.8%) in our cases of tuberculous empyema. Rupture of a tubercular cavity into pleural space and persistence of the communication constitute the mechanism of BPF in tuberculous empyema. Frank pus was aspirated in 21 cases (72%) of tuberculous empyema during initial thoracentesis out of whom Gram stain was positive in 8 patients (culture negative). The likely mechanism of bacterial superinfection in most of our tuberculous empyema patients was persistent BPF. Bacterial coinfection with tuberculous empyema has been reported in other studies.[2]

We did not proceed with pleural fluid culture for *Mycobacterium tuberculosis* because we were deterred by the high incidence of positive smears with negative cultures in LJ medium observed by others.[2,7] The frequent culture negativity despite positive smears for *M. tuberculosis* in empyema fluid is attributed to the acidic, anaerobic environment of the pleural fluid[13] and the lack of stringent laboratory conditions required for mycobacterial culture especially from purulent fluids. With the availability of sensitive culture systems like the BACTEC, culture of empyema fluid for *M. tuberculosis* has become extremely important not only for confirmation of diagnosis but also to check for drug sensitivities. There is a tendency to develop resistant organisms firstly because the antituberculous drugs may not reach their normal levels in the pleural space owing to thick fibrous pleura[14] and secondly, perhaps more importantly, due to high rates of non-/poor compliance with treatment (more than one in three patients in this study had a history of previous treatment). Undetected drug resistance may be a reason contributing to poorer outcomes in tuberculous empyema patients making attempts at culture for *M. tuberculosis* and drug-sensitivity testing desirable in future studies.

In our study, in contrast to nontuberculous empyema, tuberculous empyema patients required prolonged intercostal tube drainage and complicated surgery and had a poorer outcome. These were in keeping with other Indian studies.[2,5–8] The long duration of illness, presence of BPF, and the presence of multiple loculations were the factors associated with the adverse outcome.[2,15] However the mortality in tuberculous empyema patients (some of whom had advanced disease) in the present study (*n* = 1, 3.4%) is possibly lower than other Indian studies.[2,9] This suggests that a conservative approach with prolonged ICTD may yield a more favorable outcome in these cases of grossly compromised lung function with consequent high surgical risk. However the persistence of BPF mandates complicated surgical interference in most cases.

To conclude, tuberculosis is an important cause of empyema in the young population of our country. Tuberculous empyema patients have a protracted duration of illness, a significant incidence of BPF necessitating complicated drainage including surgical drainage and a relatively poor outcome compared to patients with nontuberculous empyema.

## References

[1] Light RW, Rhyner S, Winter N, Koleth J (2007). Parapneumonic effusions and empyema. Pleural Diseases.

[2] Malhotra P, Aggarwal AN, Agarwal R, Ray P, Gupta D, Jindal SK (2007). Clinical characteristics and outcome of empyema thoracis in 117 patients. A comparative analysis of tubercular vs. non tubercular aetiologies. Respir Med.

[3] (2003). World Health Organisation: Treatment of Tuberculosis. Guidelines for National Programmes.

[4] Maskell NA, Davies CW, Nunn AJ, Hedley EL, Gleeson FV, Miller R (2005). U.K. controlled trial of intrapleural streptokinase for pleural infection. N Engl J Med.

[5] Vardhan MV, Tewari SC, Prasad BN, Nikumb SK (1998). Empyema thoracis- study of present day clinical and etiological profile and management techniques. Ind J Tub.

[6] Goyal SP, Tandon RK, Patney NL, Mishra OP (1976). Management of tubercular empyema thoracis: A review of 53 cases. Ind J Tub.

[7] Acharya PR, Shah KV (2007). Empyema thoracis: A clinical study. Ann Thorac Med.

[8] Madan A, Sharma TN, Jain NK, Sarkar SK, Durlabhji P (1993). Tubercular empyema thoracis: A diagnostic and therapeutic problem. Indian J Chest Dis Allied Sci.

[9] Banga A, Khilnani GC, Sharma SK, Dey AB, Wig N, Banga N (2004). A study of empyema thoracis and role of intrapleural streptokinase in its management. BMC Infect Dis.

[10] (2002). World Health Organisation, TB Control. WHO Report 2002, Country Profile India.

[11] Begamy T (2000). Thoracic empyema. Is its microbiology changing?. Pul Rev Com.

[12] Alfageme I, Munoz F, Pena N, Umbria S (1993). Empyema of the thorax in adults.Etiology, microbiologic findings, and management. Chest.

[13] Nolte FS, Metchok B, Murray PR, Baron EJ, Pfaller MA, Tenver FC, Yolken RH (1995). Mycobacterium Chapter 31. Manual of clinical microbiology.

[14] Iseman MD, Madsen LA (1991). Chronic tuberculous empyema with bronchopleural fistula resulting in treatment failure and progressive drug resistance. Chest.

[15] LeMeme GP, Strange C, Sahn SA (1995). Empyema thoracis.Therapeutic management and outcome. Chest.

